# Programmed Chemotherapy for Patients with Metastatic Unresectable Gastric Cancer

**DOI:** 10.1371/journal.pone.0038652

**Published:** 2012-06-26

**Authors:** Masataka Shinoda, Takafumi Ando, Emad M. El-Omar, Hitomi Takashi, Takahisa Suzuki, Mutsumi Murayama, Kazuhiro Morise, Hidemi Goto

**Affiliations:** 1 Department of Gastroenterology, Nagoya University Graduate School of Medicine, Nagoya, Japan; 2 Department of Gastroenterology, Toyota Memorial Hospital, Toyota, Japan; 3 Department of Medicine and Therapeutics, University of Aberdeen, Aberdeen, United Kingdom; Vanderbilt University Medical Center, United States of America

## Abstract

**Background:**

Recent advances in the treatment of metastatic unresectable gastric cancers (MGC) include the development of new antitumor drugs and new regimens for their use. However, the selection of individually designed regimens by gastric cancer (GC) subtype remains problematic. Here, we investigated the clinical usefulness of programmed chemotherapy.

**Methodology/Principal Findings:**

MGC patients were classified into three groups by clinical condition. We implemented a chemotherapy program consisting of S-1 combination regimens. Median survival time (MST) of level 1 patients was 416 days (95% CI: 313–506 days), with an overall response rate of 47%. MSTs of level 2 and 3 patients were 208 (95% CI: 153–287 days) and 95 days (95% CI: 28–136 days), respectively. Grade 3–4 toxicities were neutropenia in 12% and anorexia in 6%. All treatment- related toxicities were resolved, and no treatment-related deaths occurred.

**Conclusions/Significance:**

This program provided reasonable selection of case-matching regimens and may improve the survival of patients with MGC. Further, it may represent the first clinical tool to provide efficient chemotherapy course selection for MGC. Ongoing analysis of newly developed drugs and regimens will allow the efficacy of this chemotherapy program to be improved.

## Introduction

Despite improvements in diagnostic and therapeutic methods, GC remains a major cause of death worldwide. Since its discovery by Heidelberger et al in 1957, 5-FU, an antimetabolite with strong time dependency, has been used in Japan as the gold standard drug for patients with advanced GC [1]. Because no other regimen provides better overall survival (OS), 5-FU alone has long been used as the standard arm in randomized control studies [2].

Recent advances in the treatment of MGC have seen the introduction of a new anticancer agent, S-1. This drug, a novel oral fluoropyrimidine developed from a theoretical basis which combines tegafur (5-FU derivative), gimeracil, and oteracil [3], is now changing the course of chemotherapy for MGC in Japan [4], [5]. Recent studies have shown synergistic antitumor effects of S-1 with CDDP [6], [7], PTX [8]–[10], and CPT-11 [11]–[13]. Based on evidence from the JCOG9912 and SPIRITS trials [14], S-1 has now replaced 5-FU, and combination regimens including it are now widely used in the treatment of GC in Japan [15].

Nevertheless, the selection of case-matching regimens for individual patients remains problematic. Response to chemotherapy varies from person to person, and many patients receive treatment which is suboptimum or even ineffective. The selection of regimens to manage these difficult cases is hampered by a lack of suitable guidelines. Here, we conducted a chemotherapy program that may represent a useful clinical tool in the selection of chemotherapy courses for MGC.

## Methods

### Objectives

**Figure 1:**
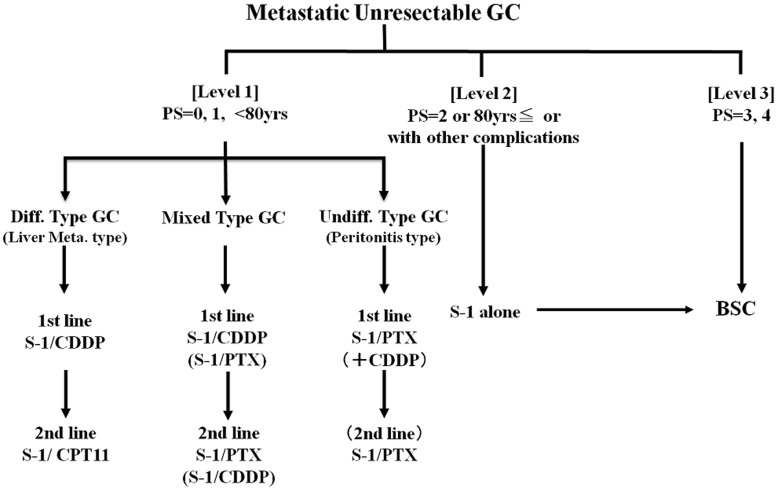
Programmed chemotherapy for patients with MGC.

The results of Japanese phase II studies indicate that sensitivity to anticancer drugs for GC differs by cellular type and GC characteristics [16]. For example, CPT-11 is more sensitive to differentiated than undifferentiated cell-type GC [17], whereas PTX is conversely more sensitive to undifferentiated than differentiated GC [18], [19]. Based on these findings, and in consideration of individual clinical conditions, we implemented a chemotherapy program consisting of S-1 combination regimens (Figure 1).

### Participants and Inclusion Criteria

From April 2004 to June 2007, 77 patients underwent treatment for MGC at our hospital, of whom 34 were classified as level 1 and treated with programmed S-1 combination regimens according to GC subtype (Table 1), 21 were classified as level 2 and treated with S-1 alone, and 22 were classified as level 3 and treated with best supportive care (BSC) alone. All patients had histological evidence of unresectable MGC, cancer stage IV including peritoneal dissemination, liver metastasis, or distant metastasis. Other inclusion criteria for the class of level 1or 2 included adequate bone marrow function (neutrophil count ≥1,500/µ l, platelet count ≥100,000/µ l and hemoglobin ≥8.0 g/dl); adequate liver function (serum bilirubin level ≤2.0 mg/dl and serum transaminase level ≤2.5×ULN (upper limits of normal)); and adequate renal function (serum creatinine level ≤1.2 mg/dl).

**Table 1:** Results of programmed chemotherapy for patients with MGC.

patients	Treatment		PR/CR	MST
level 1 (n = 34)	1^st^ line	2^nd^ line		
deifferentiated type(n = 10)	S-1+CDDP	S-1+CPT11	5/1	460days
undifferentiated type(n = 18)	S-1+PTX(+CDDP)	(S-1+PTX)	7/0	395days
mixed type (n = 6)	S-1+CDDP	S-1+PTX	2/1	481days
	S-1+PTX	S-1+CDDP		
Total	S-1 combinations		14/2	416days
level 2 (n = 21)	S-1 alone		2/1	208days
level 3 (n = 22)	Best supportive care		–	95days

PR = partial response, CR = complete response, MST = median survival time.

### Evaluations

The primary endpoint was the response rate and the secondary endpoint was based on the toxicity and OS. The clinical response was assessed according to the Response Evaluation Criteria in Solid Tumors (RECIST) [20]. In the CR case, disappearance of all endoscopic and radiographic evidence of tumor was confirmed for a minimum of 4 weeks. The therapeutic toxicity was evaluated according to the National Cancer Institute Common Toxicity Criteria version 2.0 [21]. The survival time was evaluated at Jan 16, 2010 and was analyzed by Kaplan-Meier method.

### Description of Procedures or Investigations undertaken

First, we classified patients into three groups on the basis of clinical condition. Patients aged less than 80 years with a performance status (PS) 0–1 were classified as level 1; these level 1 patients were then further classified into three subgroups according to the pathologic features of GC. Patients aged 80 years and older or with PS 2 were classified as level 2 and treated with S-1 alone; and those with severe complications or with PS 3 or 4 were classified as level 3 and treated with BSC alone. Using this program, we were easily able to select suitable first- and second-line regimens for patients with MGC. In each 28-day cycle, patients received S-1 (80 mg/sq m/day, day 1–14), CDDP (70 mg/sq m, day 8), PTX (100 mg/sq m, day 1), and CPT-11 (100 mg/sq m, day 1, 15; day 15 was skipped with grade 2 toxicity); or, in the case of CDDP plus PTX plus S-1, they received S-1 (80 mg/sq m/day, day 1–14), PTX (120 mg/sq m, day 1) and CDDP (60 mg/sq m, day 14). One chemo-treatment strategy for MGC is to set strictly scheduled chemotherapy as second-line treatment, with no pause during the transition from first-line treatment. Accordingly, we continued administering S-1 as long as the condition of the patient was not evaluated as level 3, on the basis that S-1 is an antimetabolite with strong time dependency which preserves patient quality of life (QOL) even in the case of a progressive disease (PD).

### Dose Modifications

Dose adjustments were made if grade 3–4 toxicity was seen. If grade 3–4 toxicity was present during a 4-week cycle, the administration of every anticancer drug was stopped in that cycle. In the next cycle when toxicity resolved, CDDP, PTX or CPT-11 was reduced by 20%, while S-1 was not reduced. The dose of S-1 was reduced by 30% when renal toxicity was seen (serum creatinine level ≥1.2).

### Ethics

The study was carried out in accordance with the Declaration of Helsinki. The ethics committee of cancer board, Toyota Memorial Hospital approved all of the regimens used in the program. Written informed consent was obtained from all patients prior to participation, some after visiting another hospital to receive a second opinion. The study started before Ottawa statement and had no compulsory registration of clinic trials.

## Results

### Response to Treatment

**Figure 2:**
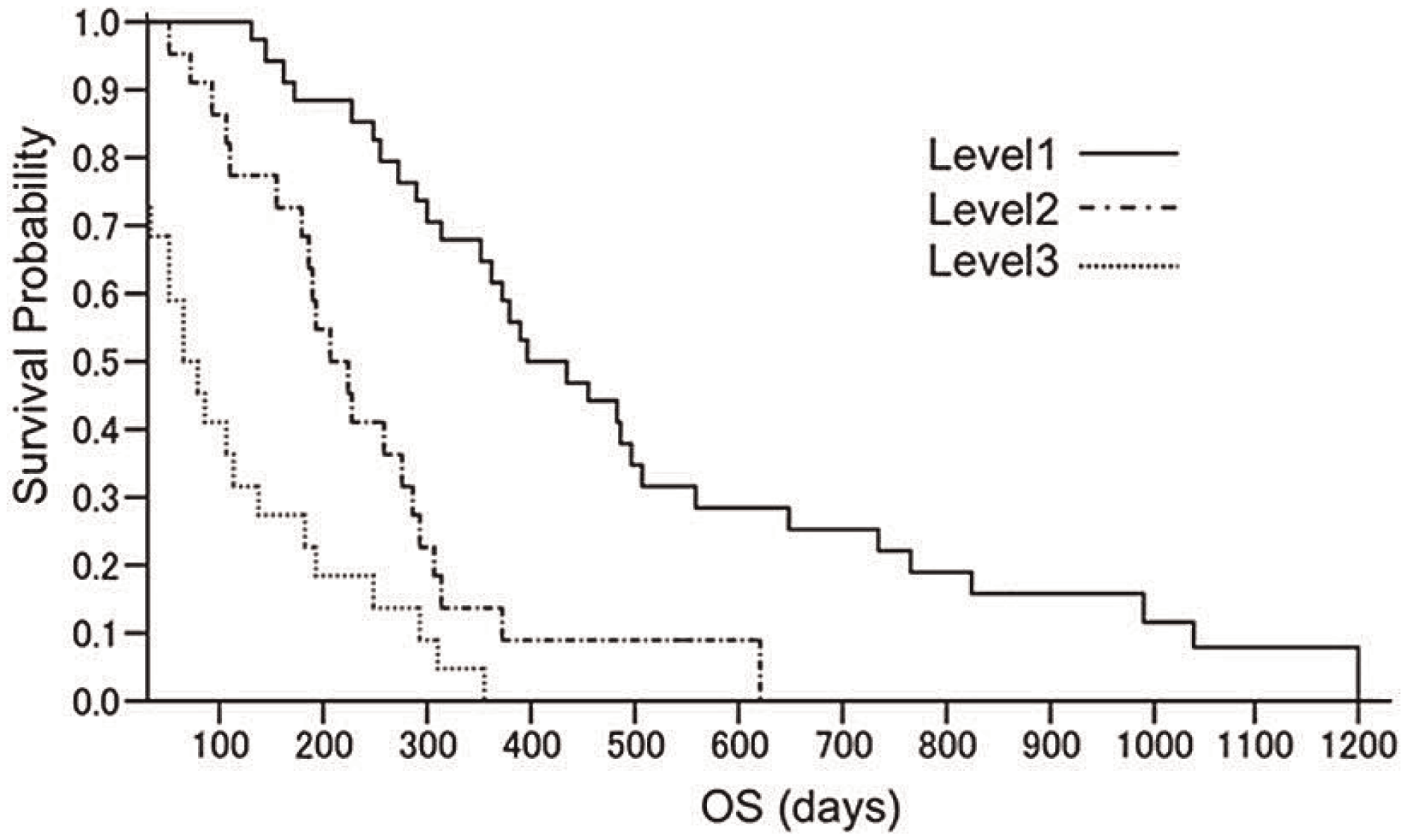
Survival curve of Level 1, Level 2 and Level 3: OS = Overall Survival.

**Figure 3:**
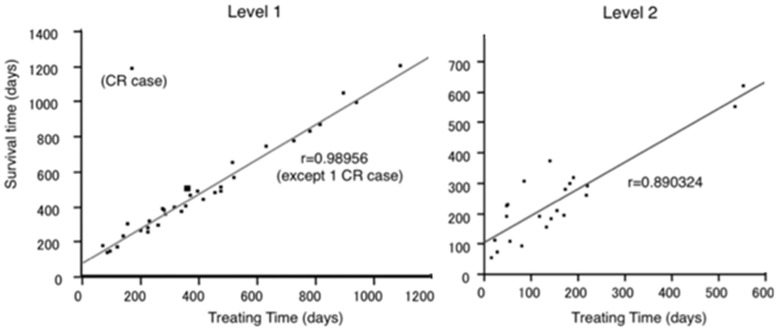
Correlation between treatment time and OS. A good correlation was seen between treatment time and survival time.

The survival curve of each level is shown in Figure 2. Median survival time (MST) of level 1 patients was 416 days (95% CI: 313–506 days), with an overall response rate of 47%. MSTs of level 2 and 3 patients were 208 (95% CI: 153–287 days) and 95 days (95% CI: 28–136 days), respectively. With regard to the outcome of level 1 patients, all 3 subgroups showed an MST of 13 months or more (Table 1). Further, the relationship between treatment time and OS correlated well, except in one complete response (CR) case (Figure 3).

### Adverse Events

Grade 3–4 toxicities were neutropenia in 12% and anorexia in 6%. All treatment- related toxicities were resolved, and no treatment-related deaths occurred.

### Representative Cases

**Figure 4:**
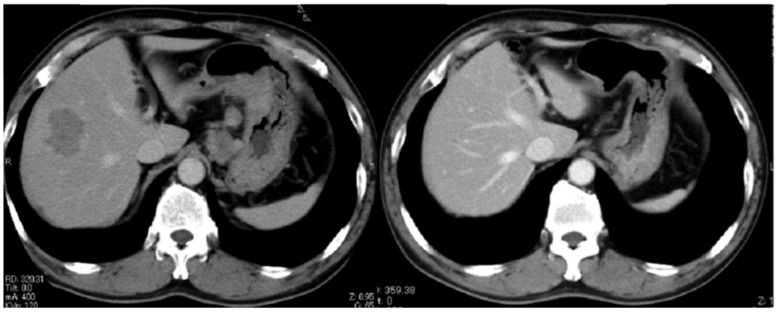
CT of Case 1, before (left; 02/08/’05) and after (right; 19/02/’09) the programmed chemotherapy. 60 yrs old male with melena was introduced to our hospital. He had differentiated-type MGC with liver metastasis and local lymph metastases. He was classified as level 1 and was treated with S-1 plus CDDP combination for 6 cools. CR was confirmed radiologically.

Case 1: A 60-year-old male was admitted with melena and diagnosed with differentiated-type MGC with liver metastasis and local lymph node metastasis. He was classified as level 1 and treated with S-1 plus CDDP combination for four cycles. CR was confirmed radiologically (Figure 4) and endoscopically. Two years later, he was informed of local lymphadenopathy but declined surgery. CR was reacquired after an additional two cycles of S-1 plus CDDP. In this case, strictly scheduled chemotherapy provided a maximum anti-tumor effect.

**Figure 5:**
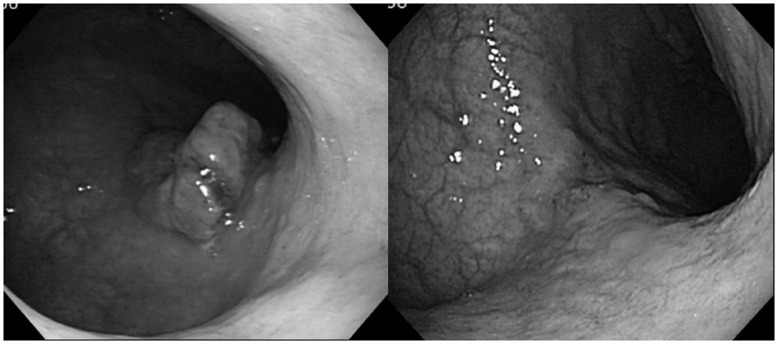
Endoscopic features of Case 2. PR was confirmed in Case 2 by endoscopic features before (left; 21/02/’07) and after (right; 29/06/’07) programmed chemotherapy.

Case 2: A 70-year-old male diagnosed with differentiated-type MGC with massive liver metastasis was treated with S-1 plus CDDP combination for two cycles. Following radiological confirmation of PD, he was treated with four cycles of S-1 plus CPT-11, on the basis that even when one S-1 combination therapy is evaluated as PD, another might be effective. We therefore selected the next S-1 combination in accordance with the program for this patient, and he finally achieved partial response (PR) (Figure 5).

**Figure 6:**
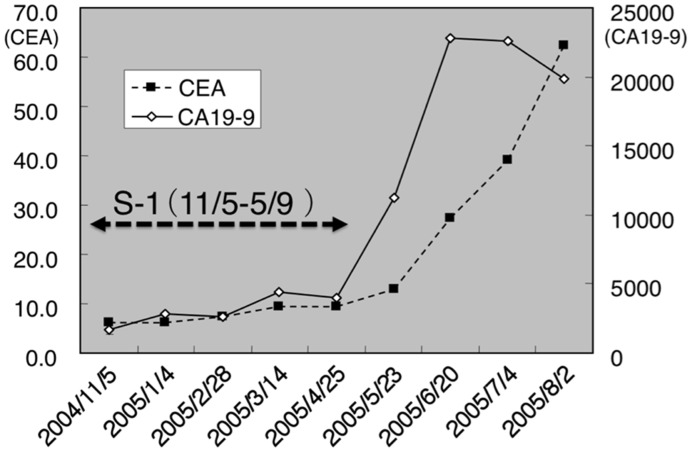
The course of tumor markers in Case 3. CEA and CA19-9 increased markedly when we stopped giving S-1, suggesting that S-1 was still effective to control the case.

Case 3: A 82 year old female presented with anorexia and anemia. She had undifferentiated-type MGC and her condition was classified as level 2. She was given a blood transfusion and treatment was started with S-1 alone on November 5, 2004. She died from liver metastasis, while pathological CR was found by stomach dissection. Because she refused further chemotherapy, we stopped giving S-1 on May 9, 2005. Her tumor markers increased markedly after we stopped S-1 treatment, suggesting that S-1 should have been effective for controlling this case (Figure 6). We propose that S-1 should be given continuously, unless it is toxic.

## Discussion

The aim of this study was to evaluate the usefulness of programmed chemotherapy for patients with MGC. Results showed the value of this strategy for the selection of case-matching regimens for patients with MGC. Additional data will confirm this program, and provide for its ongoing refinement.

While a substantial portion of the more than 50,000 deaths annually from GC in Japan are due to MGC, response to chemotherapy in these patients varies widely, and some in fact receive ineffective treatment. Against this background, recent studies have shown synergistic antitumor effects of S-1, a new anticancer agent which has changed the course of chemotherapy for MGC in Japan, and CDDP, CPT-11, and taxenes [6]–[15]. Novel approaches to patient selection and the individual design of chemotherapy regimens are thus urgently required, but treatment course programs for patients with MGC have not been reported. We therefore conducted a prudent initial investigation of chemotherapy programs composed of fixed S-1 combination regimens.

Evidence from the JCOG 9912 and SPIRITS trials [14] has established the S-1 plus CDDP combination as the standard first-line regime for MGC in Japan. The consensus for this combination is based on evidence from the SPIRITS trial, which confirmed OS prolongation as the primary endpoint. However, these trials also allowed second- or third-line treatments on ethical grounds. OS is actually an outcome of the aggregate of all therapies, including first-, second-, and third-line treatment with sequential BSC. In some studies, completely ineffectual first-line therapy might be deemed as markedly effective even when it is in fact second- or third-line therapy which rescues the patient. For example, our case 2 patient might have died prematurely from liver failure with the standard first-line treatment of S-1 plus CDDP, but actually survived for 496 days with the second-line treatment of S-1 plus CPT-11. We evaluated this case as PD with S-1 plus CDDP, and PR with subsequent S-1 plus CPT-11. Because sensitivity to anticancer drugs appears to differ by cellular type and GC characteristics, outcomes will be optimized by the selection of an individually designed regimen or change to a suitable second-line regimen.

In Japan, the new anticancer agent S-1 has replaced 5-FU for MGC and is now considered a key drug in this use, with many combination regimens which incorporate it now in use [8], [11], [15]. Many clinical trials of combination chemotherapy with S-1, CDDP, CPT-11, and PTX have obtained response rates higher than 30%. With regard to effectiveness, CDDP or CPT-11 is reported to be effective for differentiated-type GC [16], [17], and PTX for undifferentiated-type GC [16], [18], [19]. Following these Japanese phase II study results, we conducted a chemotherapy program and investigated the effectiveness of this program. Prognosis should be improved by the timely selection and ongoing evaluation of individually matched regimens for patients with various GC subtypes. A second important point is the construction of effective second- and third-line regimens which extend remission and survival. We selected S-1-based regimens in our program on the basis of their time-dependent anti-tumor effects with lower toxicity and the convenience of oral delivery. Recently, S-1-based sequential chemotherapy as second-line treatment was reported to prolong OS with less toxicity than other second-line regimens which did not include S-1 [22].

In the present study, MSTs for levels 2 and 3 were 208 days (95% CI: 153–287 days) and 95 days (95% CI: 28–136 days), respectively, while that of level 1 was 416 days (95% CI: 313–506 days), giving an overall response rate of 47%. These results indicate the clinical value of this program as a tool for the selection of case-matching regimens for patients with MGC. In addition, a significant correlation was seen between treatment time and survival. Our strategy of giving S-1 even to PD patients with PS 0 to 2 appears effective, and we recommend that administration be given continuously to preserve QOL, provided toxicity is acceptable.

Our program provides the efficient sequential use of anticancer drugs for patients with MGC. We consider that this program may provide superior selection of case-matching treatment to those previously reported and may further improve the survival of patients with MGC. Ongoing analysis of newly developed drugs and regimens will allow further refinement of the program.

This program was a reasonable clinical tool for selecting case-matching regimens for patients with MGC. Additional data will allow the efficacy of the program to be confirmed, and provide for ongoing refinement. Further clinical trials should investigate the sequential use of anticancer drugs, molecular target drugs, and surgery, as well as combinations thereof.
